# Intrinsic variability in Pv, RRP size, Ca^2+^ channel repertoire, and presynaptic potentiation in individual synaptic boutons

**DOI:** 10.3389/fnsyn.2012.00009

**Published:** 2013-01-11

**Authors:** Pablo Ariel, Michael B. Hoppa, Timothy A. Ryan

**Affiliations:** ^1^Department of Biochemistry, Weill Cornell Medical CollegeNew York, NY, USA; ^2^David Rockefeller Graduate Program, The Rockefeller UniversityNew York, NY, USA

**Keywords:** synapse, exocytosis, release probability, pHluorin, readily releasable pool, imaging

## Abstract

The strength of individual synaptic contacts is considered a key modulator of information flow across circuits. Presynaptically the strength can be parsed into two key parameters: the size of the readily releasable pool (RRP) and the probability that a vesicle in that pool will undergo exocytosis when an action potential fires (*Pv*). How these variables are controlled and the degree to which they vary across individual nerve terminals is crucial to understand synaptic plasticity within neural circuits. Here we report robust measurements of these parameters in rat hippocampal neurons and their variability across populations of individual synapses. We explore the diversity of presynaptic Ca^2+^ channel repertoires and evaluate their effect on synaptic strength at single boutons. Finally, we study the degree to which synapses can be differentially modified by a known potentiator of presynaptic function, forskolin. Our experiments revealed that both *Pv* and RRP spanned a large range, even for synapses made by the same axon, demonstrating that presynaptic efficacy is governed locally at the single synapse level. Synapses varied greatly in their dependence on N or P/Q type Ca^2+^ channels for neurotransmission, but there was no association between specific channel repertoires and synaptic efficacy. Increasing cAMP concentration using forskolin enhanced synaptic transmission in a Ca^2+^-independent manner that was inversely related with a synapse's initial *Pv*, and independent of its RRP size. We propose a simple model based on the relationship between *Pv* and calcium entry that can account for the variable potentiation of synapses based on initial probability of vesicle fusion.

## Introduction

Synapses made by the same axon can have very different neurotransmitter release characteristics. There are reports of substantial variation in the probability of neurotransmitter release (*Pr*) using many techniques (Rosenmund et al., 1993; Murthy et al., 1997; Branco et al., 2008, 2010; Granseth and Lagnado, 2008; Matz et al., 2010; De Jong et al., 2012). Important influences on *Pr* at each synapse are the identity of target neurons (Markram et al., 1998; Reyes et al., 1998; Sylwestrak and Ghosh, 2012), the activity level and position on dendrites being contacted (Branco et al., 2008; De Jong et al., 2012), and the GABA concentration in the local microenvironment (Laviv et al., 2010). However, how these influences translate into different *Pr* values for synapses along the same axon is largely unknown.

*Pr* gives a measure of the all-or-none behavior of synapses, i.e., the probability that at least one synaptic vesicle will fuse following an action potential (AP). Two main building blocks influence *Pr*. First, the number of vesicles, *n*, at the active zone in a biochemical state that only requires calcium entry to trigger exocytosis [also known as the readily releasable pool (RRP)]. Second, the probability that each vesicle will fuse with the membrane in response to an AP (*Pv*). This parameter describes in part the likelihood that calcium influx within the active zone satisfactorily binds the calcium sensor to trigger exocytosis. In a binomial model of neurotransmitter release (Schneggenburger et al., 2002) the average number of vesicles that fuse in response to an AP will be the product of *n* and *Pv*, whereas *Pr* will be:

(1) $$\text{Pr}=1-{\left(1-Pv\right)}^{n}$$

Various factors can influence *Pv* and RRP size independently (Ariel and Ryan, 2012). Although previous studies examined *Pr* variation across individual synapses, most measures of *Pv* and RRP are carried out only for ensembles of synapses (for example, Jockusch et al., 2007; Weston et al., 2011). A recent study explored *Pv* and RRP size at individual boutons of neurons in hippocampal cultures, and found substantial variation in both parameters, even for synapses made by the same axon (Ermolyuk et al., 2012). *Pv* correlated with the amount of presynaptic Ca^2+^ influx, suggesting this is a key control variable that sets differences in efficacy between synapses.

Given that there are multiple Ca^2+^ channel subtypes that contribute to the Ca^2+^ influx necessary for neurotransmitter release, we wondered whether different repertoires of Ca^2+^ channels might be associated with synapses of different strengths. There is evidence that individual boutons can vary substantially in their dependence on N or P/Q-type channels for exocytosis (Reuter, 1995). In young neurons grown in autaptic cultures, there was no correlation between *Pr* and the distribution of N or P/Q-type channels (Reid et al., 1997). However, that study did not directly examine individual synapses, and it is unclear whether its results can be extrapolated to more mature synapses. For example, during maturation of the calyx of Held synapse, P/Q-type channels become the main drivers of neurotransmitter release by more closely coupling to synaptic vesicles (Fedchyshyn and Wang, 2005). Thus, the role of Ca^2+^ channel heterogeneity across small individual synapses remains unclear.

In addition to differing in baseline neurotransmitter release properties, individual synapses can vary in how they are modulated. For example, type III metabotropic glutamate receptor 7 (mGluR7) is specifically enriched in presynaptic terminals that contact interneuron targets (Shigemoto et al., 1996; Lujan et al., 1997). Consequently, glutamate release from individual CA3 pyramidal cells is inhibited by type III mGluR agonists at synapses onto interneurons, but not pyramidal cells (Scanziani et al., 1998). Another example is the negative modulation of *Pr* by GABA_B_ receptors. In young hippocampal neurons in autaptic culture, the agonist baclofen does not affect a subset of synapses with high *Pr*, suggesting receptors are heterogeneously distributed in presynaptic terminals of the same axon (Rosenmund et al., 1993). Beyond these examples, the issue of presynaptic synapse-specific modulation has not been extensively studied.

Here we extended previously developed optical measures of *Pv* and RRP size (Ariel and Ryan, 2010) to determine how these parameters vary across populations of individual nerve terminals. Subsequently, we studied whether different repertoires of N and P/Q type Ca^2+^ channels were predictive of synaptic strength. Finally, we examined how *Pv* and RRP size are impacted by direct activation of adenylyl cyclase by forskolin, a potentiator of presynaptic function, to determine the variability of how individual synapses can be potentiated.

## Results

### Measurements of *Pv* and RRP size at individual synapses

To obtain reliable estimates of *Pv* and RRP size at individual synapses we extended a previously developed optical method based on the vesicular glutamate transporter fused to the pH-sensitive GFP pHluorin (vG-pH, Ariel and Ryan, 2010). The approach makes use of the single AP-sensitivity of vG-pH responses that can be averaged over many trials to obtain robust single bouton AP response estimates (see below). These are compared to measurements of the RRP size obtained by examining the kinetics and amplitudes of exocytosis in response to high-frequency (100 Hz) AP firing. To improve the robustness of RRP measurements at single synapses we modified our previous protocol for ensembles of synapses (Ariel and Ryan, 2010), enhancing the ability to detect a plateau phase indicative of RRP depletion in the kinetics of exocytosis during high frequency AP firing (Materials and Methods).

One of the main issues to consider when measuring responses from individual synapses is that potentially large statistical fluctuations are expected from a binomial process with a relatively low *Pv* and *n* (*n* = number of vesicles in the RRP). Our experiments consist of several trials where the stimulus is a single AP. These responses are averaged for each synapse and compared to the corresponding estimate of RRP size, giving a measure of *Pv*. If we assume that a binomial process with parameters *Pv* and *n* fully describes the behavior of a synapse during this kind of experiment, we can ask how much the *measured Pv* will fluctuate around the *real Pv*. The relative size of those fluctuations will depend on *Pv*, *n*, and the number of trials (*k*) in the experiment. From simple binomial statistics, the expected coefficient of variation (CV) in an estimate of *Pv* will be:

(2) $$\text{CV}=\sqrt{\frac{\left(1-Pv\right)}{k\times Pv\times n}}$$

Note that these fluctuations are unavoidable and would be present even if using an idealized detection system without any noise. The main way to minimize their effect in any given experiment is to perform as many trials as possible. For example, an experiment to estimate *Pv* in single synapses with an expected CV of 15% would require 100 trials, assuming *Pv* = 0.1 and *n* = 4 [our averages under standard conditions, see Ariel and Ryan (2010)]. Obtaining such a large number of measurements is problematic because it requires long experiments and total cumulative exposures to laser illumination that can be detrimental to cell health. By extension, it is doubly difficult to execute a baseline measurement, follow it with a pharmacological application and measure the effect of that intervention. Furthermore, because our calculation only illustrates the CV of an *average* synapse, we expect many measurements—in lower *Pv* synapses—to be subject to larger fluctuations. For all of these reasons, we chose to increase the concentration of extracellular Ca^2+^ ions to 4 mM. Based on our previous results (Ariel and Ryan, 2010) we expected this would raise *Pv* to approximately 0.35. Using Equation 2, to measure *Pv* with an expected CV of 15% under these conditions requires only 21 trials. This allows relatively shorter experiments where maintaining cell viability is feasible.

**Figure 1:**
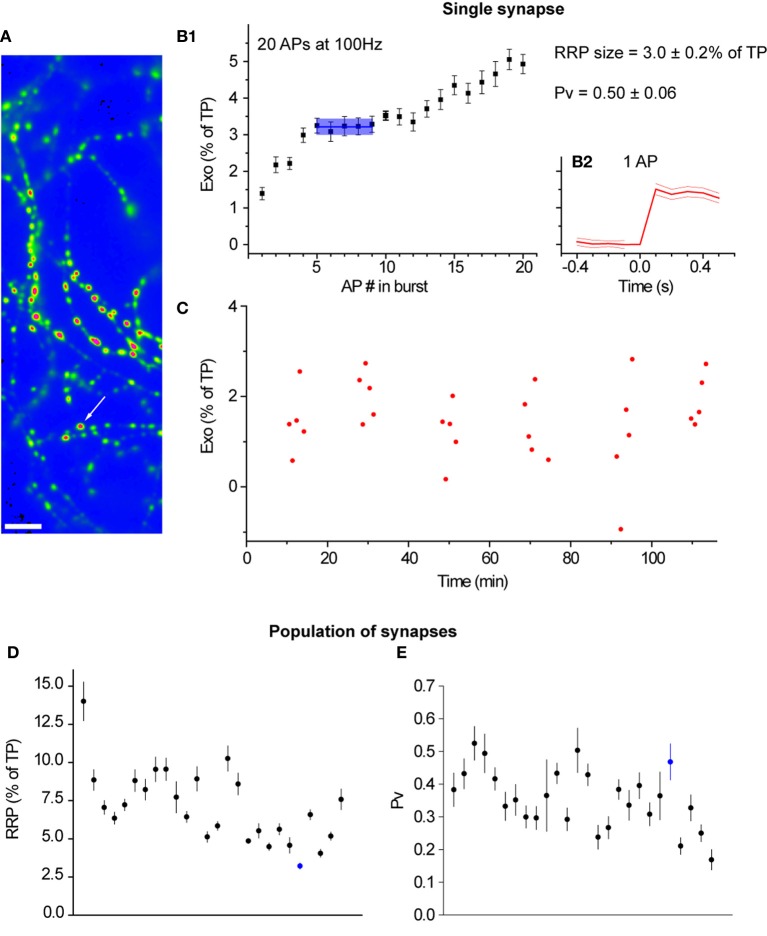
***Pv* and RRP size can be measured precisely at many individual synapses in parallel. (A)** Field of boutons in a representative experiment. The image is the difference in fluorescence before and after the application of 50 mM NH_4_Cl and is smoothed for presentation purposes only. The arrow marks the synapse shown in detail in the rest of the figure. Scale bar = 5 μm. **(B1)** Cumulative exocytosis in response to 20 APs at 100 Hz for the indicated synapse (*n* = 12 trials). The blue line indicates the RRP size. The light blue shading indicates the region where a plateau, indicative of exhaustion of the RRP, was detected using our methods, along with the SE in the RRP size (see Materials and Methods). **(B2)** Exocytosis in response to a single action potential. The thick red line indicates an average over 30 trials. The thin red lines show the SE of this average. The vertical scale is the same as in **(B1)** and is aligned with that panel for convenience. **(C)** Responses to single APs for the indicated synapse. Note the stability in the response throughout almost 2 h of imaging. **(D)** RRP size and **(E)** *Pv* respectively for 26 synapses in this experiment that passed our filtering criteria (see Materials and Methods). Each point corresponds to an individual synapse. Synapses are ordered in both panels according to their single AP responses (as fraction of TP) from highest (left) to lowest (right). The synapse marked in blue is the one analyzed in **(B1), (B2)**, and **(C)**.

To robustly estimate both *Pv* and RRP size from individual synapses, we repeated many trials where we measured the responses to both single APs (30 trials) and bursts at 100 Hz (12 trials). For each synapse, we calculated the SEs in the estimates of *Pv* and RRP size using conventional error propagation techniques (see Materials and Methods). The number of trials in our experimental design was sufficient to precisely determine *Pv* and RRP at individual synapses. In the case of RRP size, the median SE—expressed as % of TP—was 0.4%. In the case of *Pv*, the median SE was 0.06. In total, we measured *Pv* and RRP size in 410 synapses from 32 experiments (see Materials and Methods for further details). Figure 1 shows data from a representative experiment.

To check whether our RRP size results were consistent with previous measurements we estimated how many vesicles were present in the RRP. To that end we assumed that all synaptic vesicles were labeled with the vG-pH reporter and that, on average, there were 64 vesicles in the releasable pool of vesicles (Balaji and Ryan, 2007). Furthermore, we assumed that the releasable pool of vesicles constituted 60% of the total pool of labeled vesicles (Fernandez-Alfonso and Ryan, 2008). Combining these assumptions we estimated that, on average, there would be 106 labeled vesicles in each synapse. Comparing this to the average size (across all synapses) of the fluorescence response to a brief alkalinizing pulse of NH_4_Cl we estimated the fluorescence of an individual labeled synaptic vesicle. This parameter allowed us to calculate the average number of vesicles in the RRP (4.2 ± 0.1) which was in good agreement with our previous results using cell-wide measurements (Ariel and Ryan, 2010) and the number of docked vesicles observed by electron microscopy in hippocampal synapses in culture (Schikorski and Stevens, 1997). Using our estimates of *Pv* and *n* in Equation 2, the median expected CV of *Pv* measurements—due solely to statistical fluctuations—was 11% (range = 0–39%).

### Individual synapses vary considerably in *Pv* and RRP size

**Figure 2:**
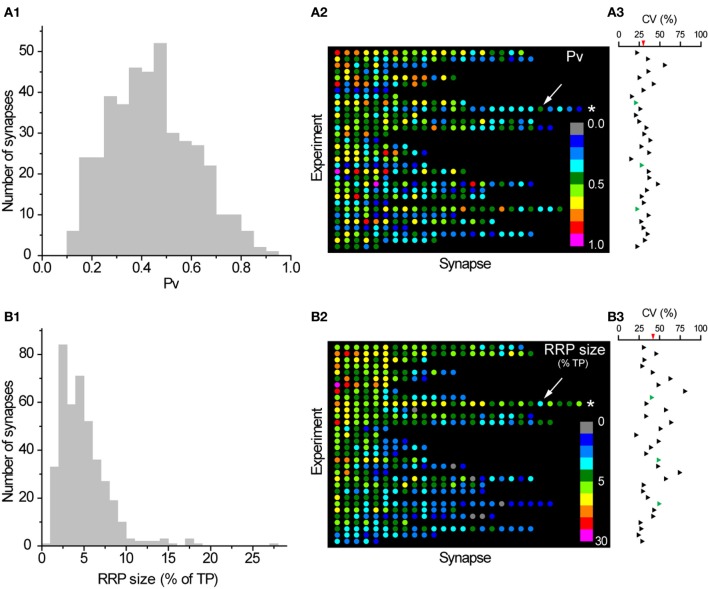
**Large variation in *Pv* and RRP size between individual synapses. (A1)** Histogram of *Pv* across synapses (a synapse with 1.1 ± 0.2 was excluded for convenience of presentation). **(A2)** Color map of *Pv* values in all synapses included in our analysis. Each row in this plot represents a single experiment and each dot within that row a single synapse from that experiment. Synapses are colored according to their *Pv* with cold colors representing low *Pv* values and warm colors representing high *Pv* values. The inset shows the *Pv* color scale. Synapses within each experiment are ranked according to their average response to a single AP (as % of TP) from left (highest) to right (lowest). Experiments are ordered according to their average synaptic response to a single AP from top (highest) to bottom (lowest). The asterisk highlights the experiment shown in Figure 1 and the arrow indicates the synapse shown in more detail in Figures (1B,C). **(A3)** The CVs in *Pv* values for each experiment are shown aligned with the corresponding row in **(A2)**. The average CV in *Pv* across experiments is shown as a red triangle in the scale bar. Three experiments where all synapses belonged to the same axon are shown in green. **(B1–B3)** Histogram, color map, and CVs within each experiment of RRP size (as % TP), analogous to **(A1–A3)**. Synapses in **(B2)** are ordered identically to **(A2)** so a dot in the equivalent position on the color maps represents the same synapse, while equivalent rows represent the same experiment. Note that the scaling of colors coding RRP sizes is logarithmic.

Confident that we had robust measures of *Pv* and RRP size at individual boutons, we studied the variability in these basic exocytosis parameters between synapses. Interestingly, we found a large degree of variability in both parameters (Figure 2). Overall the average CV across the sampled population was 39% for *Pv* and 61% for RRP size. Furthermore, a considerable amount of this variability was present between synapses in the same experiment. The average CV of *Pv* within each experiment was 30% (range: 22–56%) and 42% for RRP size (range: 21–80%). The variability in RRP size was not a consequence of our normalization to the size of the total pool of vesicles within each synapse as the CVs were still large even when we considered raw Δ*F* values of RRP size (CV_all_ = 61%; CV_within_ = 37%; range = 15–56%). It is important to note that we could not initially rule out that synapses included in our experiments belonged to axons from different somas as we frequently had more than one neuron transfected in a given dish. Thus, variation between synapses might be due to differences between the neurons that give rise to various subsets of boutons in any given imaged field. To directly obtain estimates of the variability between synapses made by the same axon we took two approaches, detailed below.

First, we used retrospective immunostaining for vG-pH to trace the entire axonal arbor in a subset of our experiments. In most cases (6/9) several transfected somas gave rise to axons that intercrossed in the imaged region such that we could not unambiguously determine that all synapses under study came from just one neuron. However, in three experiments retrospective tracing of processes proved that all imaged synapses were indeed formed by the same axon (green triangles in Figures 2A3 and 2B3). The CV of *Pv* in those experiments was 20% (*n* = 14 synapses), 27% (*n* = 10 synapses), and 22% (*n* = 24 synapses). On the other hand, the CV of RRP size was 40, 48, and 49%, respectively. This analysis shows that a large amount of variability is present in *Pv* and RRP size even between synapses made by the same axon, with the caveat that the conclusion is drawn from a small number of observations.

Second, in several cases we were able to determine unambiguously that small groups of adjacent boutons belonged to the same axonal branch. Close inspection of images taken during some experiments clearly showed isolated lengths of axon with groups of three or more boutons, arranged *en passant*. In total, this analysis revealed 64 synapses grouped in 17 axonal branches in 15 experiments. We calculated the CV in *Pv* and RRP size across boutons in each axonal branch (subsequently averaging across branches in experiments with more than one group of boutons that fulfilled the criteria). The average CV in *Pv* (across experiments) between boutons of the same axonal branch was 38%. This suggests that all of the variability in *Pv* between synapses in an experiment (CV = 30%, see above) is present even among boutons on the same axonal branch. Similarly, the average CV in RRP size (across experiments) between boutons of the same axonal branch (46%) was comparable to that of all boutons in a given experiment (42%, see above). In conclusion, synapses can differ greatly in *Pv* and RRP size, even if they are in close proximity on the same axonal branch.

### Ca^2+^ channel repertoire does not determine efficacy of neurotransmitter release at individual synapses

Having established that synapses made by the same axon could vary substantially in *Pv* and RRP size, we explored the idea that different Ca^2+^ channel subtype repertoires at individual synapses might be associated with varying efficacies of neurotransmitter release.

**Figure 3:**
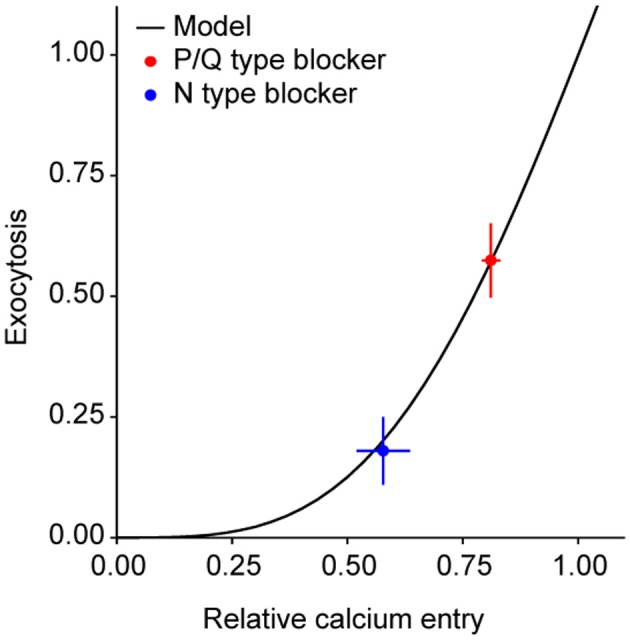
**P/Q and N-type Ca^2+^ channels do not differ in their coupling to primed synaptic vesicles.** Exocytosis as a function of the relative Ca^2+^ entry in response to 1 AP. The model is from a fit to exocytosis vs. Ca^2+^ entry data in a previous publication [Figure 2C in Ariel and Ryan (2010)]. For convenience, exocytosis and Ca^2+^ have been renormalized to the expected values for 1 AP at 4 mM extracellular Ca^2+^. Note the good agreement between the model and the data in the presence of toxins.

Before addressing this issue directly, we first determined the relative contributions of N and P/Q type Ca^2+^ channels to exocytosis in response to 1 AP in our system. Using our previously developed methods to study exocytosis in groups of synapses (Ariel and Ryan, 2010), we found that ω-agatoxin IVA (a specific blocker of P/Q type Ca^2+^ channels) caused a 43 ± 8% decrease in single AP exocytosis (*n* = 12 experiments). Conversely, selectively blocking N type Ca^2+^ channels with ω-conotoxin GVIA caused an 82 ± 7% reduction in exocytosis (*n* = 10 experiments). In principle, the larger effect on exocytosis of blocking N-type Ca^2+^ channels could be due to the presence of more of those channels, a larger current per channel, or to closer coupling between that channel type and primed vesicles. To test the last of these possibilities, we measured the effects of ω-agatoxin IVA and ω-conotoxin GVIA on Ca^2+^ entry in response to a single AP, using MgGreen-AM. Blocking P/Q type Ca^2+^ channels reduced Ca^2+^ entry by 19 ± 2% (*n* = 4 experiments) whereas blocking N-type Ca^2+^ channels caused a 42 ± 6% decrease (*n* = 5 experiments). If the coupling of each Ca^2+^ channel subtype to vesicles were the same, we would expect the reduction in Ca^2+^ entry caused by either toxin to cause a decrease in exocytosis predictable by the curve that relates exocytosis and Ca^2+^ entry under control conditions [Figure 2C in Ariel and Ryan (2010)]. However, if N type channels were closer to vesicles, there should be a larger decrease in exocytosis for a given reduction in Ca^2+^ than expected from the control curve. Thus, plotting the exocytosis and Ca^2+^ entry data in the presence of toxins and comparing it to the control curve is a test of whether the coupling between Ca^2+^ channels and primed vesicles differs according to subtype. In fact, the data obtained in the presence of either toxin agrees well with our control curve relating exocytosis and Ca^2+^ entry (Figure 3). This indicates there is no difference in coupling between P/Q and N Ca^2+^ channel subtypes. Therefore, the larger contribution of N-type Ca^2+^ channels to exocytosis might be due to larger numbers of active channels in the presynaptic membrane or a higher current per channel.

Once we had determined the relative importance of N and P/Q-type Ca^2+^ channels for exocytosis, we tested the hypothesis that different repertoires or “mixes” of Ca^2+^ channel subtypes are associated with synapses of different efficacies. To that end, in a subset of the experiments designed to measure properties at single synapses, we applied toxins specific to each Ca^2+^ channel subtype after measuring basal *Pv* and RRP size. We reasoned that if Ca^2+^ channel subtypes were differentially distributed across synapses with different efficacies, there would be a correlation between the effect of the toxins on single AP responses and *Pv*. On the other hand, *a priori* we did not expect a correlation between the effect of either toxin and RRP size.

**Figure 4:**
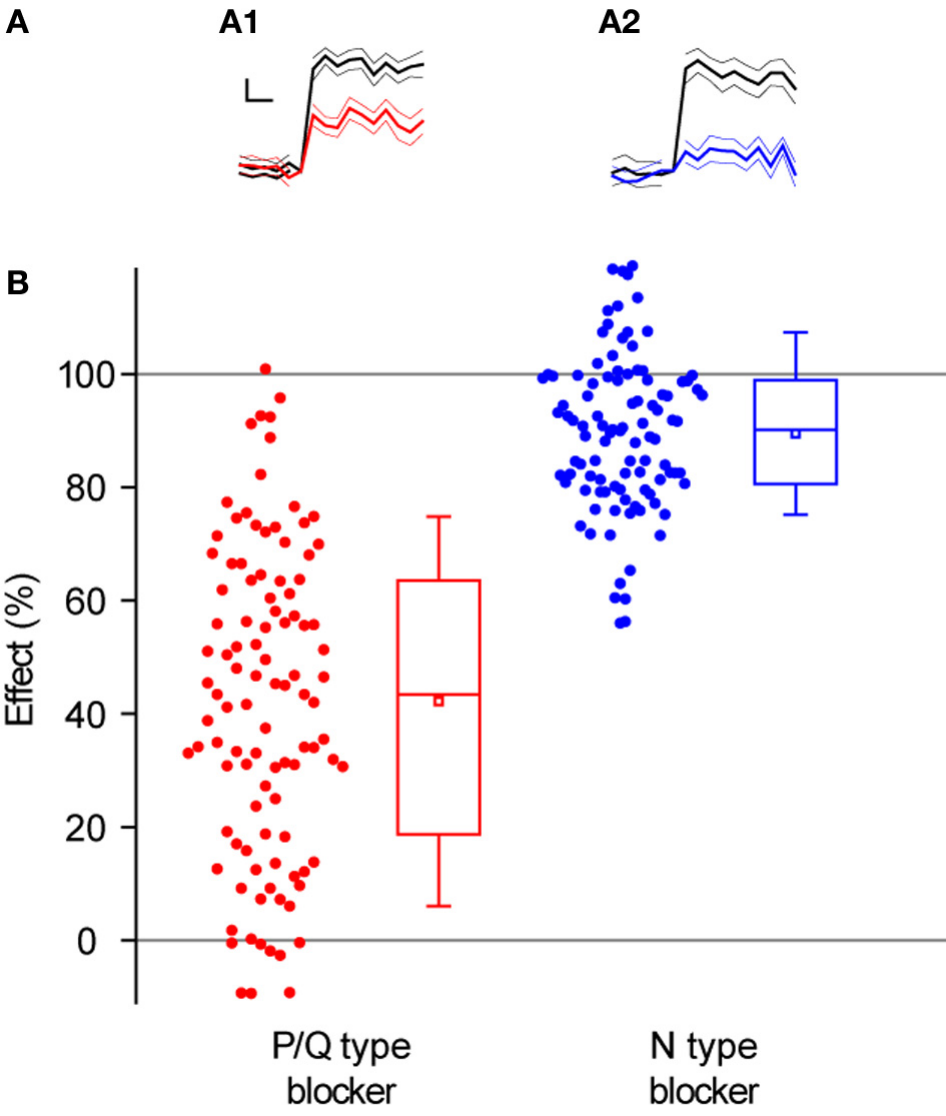
**Block of P/Q-type Ca^2+^ channels leads to weaker and more variable decrease of exocytosis than block of N-type Ca^2+^ channels. (A)** Representative single synapse responses to 1 AP stimulus before and after the application of toxins that block Ca^2+^ channel subtypes. **(A1)** Response to 1 AP before (black) and after (red) applying ω-agatoxin IVA (effect of toxin = 45 ± 12%). **(A2)** Response to 1 AP before (black) and after (blue) applying of ω-conotoxin GVIA (effect of toxin = 81 ± 11%). Each trace in **(A1)** and **(A2)** is an average of 30 trials with the thinner lines representing the SEs. Traces are normalized to the size of the response before applying the corresponding toxin and shown on the same scale. Scale bar: 0.2 s and 20% of pre-toxin response. **(B)** Effect of P/Q and N-type Ca^2+^ channel blockers across all synapses. The effect of a toxin is defined as the percentage decrease in single AP responses. Each dot corresponds to one synapse. Box whisker plots show the median (line), mean (point), 25–75 percentile (box), and 10–90 percentile (whisker) ranges (*n* = 103 synapses from 9 experiments for P/Q-type blocker, 101 synapses from 8 experiments for N-type blocker).

**Figure 5:**
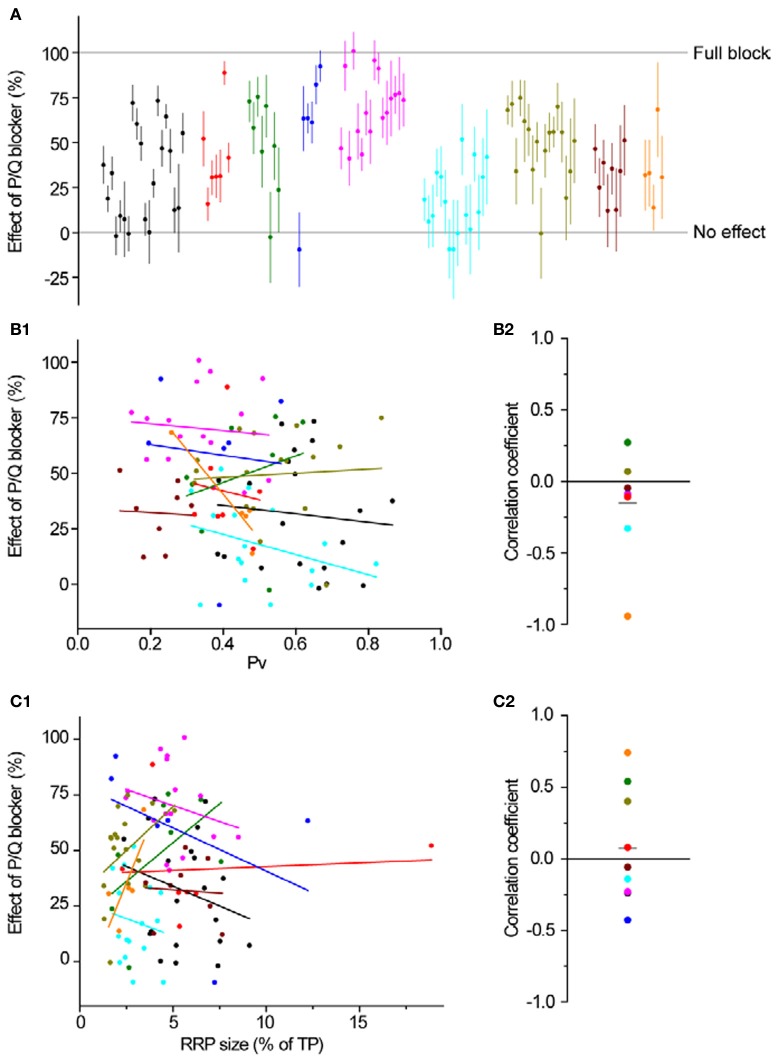
**P/Q-type Ca^2+^ channel distribution is independent of *Pv* and RRP size. (A)** The effect of ω-agatoxin IVA on exocytosis in response to a single AP. Different colors represent individual experiments, while each dot shows the effect of the blocker on a single synapse (with its SE). Experiments are ordered according to their responses to an individual AP from highest (left) to lowest (right). Within an experiment, synapses are also sorted from most (left) to least responsive (right). **(B1,B2)** Effect of a P/Q blocker is independent of *Pv*. **(B1)** Each dot is a synapse and the coloring scheme is the same as in **(A)**. Lines represent best fits for each experiment, colored accordingly. **(B2)** Correlation coefficients of the effect of P/Q block with *Pv* for were not significantly different from 0 (one-sample, two-tailed *t*-test against null hypothesis μ = 0, *P* = 0.21, *t*-value = −1.36). Each dot represents the value of the correlation coefficient for 1 experiment, with colors consistent with the rest of the figure. The gray line represents the average correlation coefficient across experiments. **(C1,C2)** Effect of a P/Q blocker is independent of RRP size. Coloring and symbols are analogous to **(B1)** and **(B2)**. Correlation coefficients of the effect of P/Q block with RRP size for each experiment were not significantly different from 0 (one-sample, two-tailed *t*-test against null hypothesis μ = 0, *P* = 0.60, *t*-value = 0.55).

**Figure 6:**
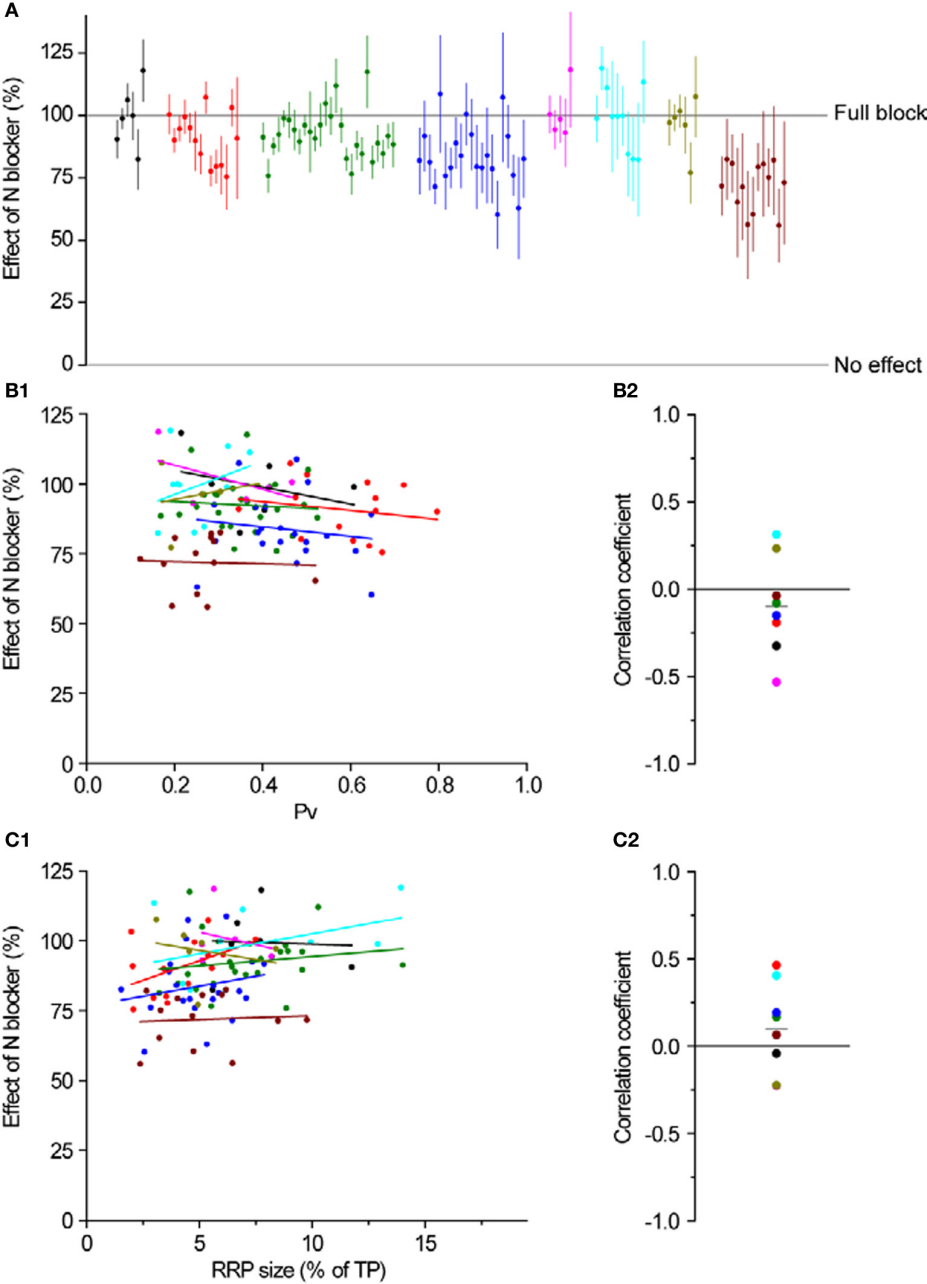
**N-type Ca^2+^ channel distribution is independent of *Pv* and RRP size. (A)** The effect of ω-conotoxin GVIA on exocytosis in response to a single AP. Different colors represent individual experiments, while each dot shows the effect of the blocker on a single synapse (with its SE). Experiments are ordered according to their responses to an individual AP from highest (left) to lowest (right). Within an experiment, synapses are also sorted from most (left) to least responsive (right). **(B1,B2)** Effect of an N blocker is independent of *Pv*. **(B1)** Each dot is a synapse and the coloring scheme is the same as in **(A)**. Lines represent best fits for each experiment, colored accordingly. **(B2)** Correlation coefficients of the effect of N block with *Pv* for each experiment were not significantly different from 0 (one-sample, two-tailed *t*-test against null hypothesis μ = 0, *P* = 0.36, *t*-value = −0.98). Each dot represents the value of the correlation coefficient for 1 experiment, with colors consistent with the rest of the figure. The gray line represents the average correlation coefficient across experiments. **(C1,C2)** Effect of an N blocker is independent of RRP size. Coloring and symbols are analogous to **(B1)** and **(B2)**. Correlation coefficients of the effect of N block with RRP size for each experiment were not significantly different from 0 (one-sample, two-tailed *t*-test against null hypothesis μ = 0, *P* = 0.32, *t*-value = 1.08).

Overall, blocking N-type Ca^2+^ channels led to a larger decrease in single AP responses at individual synapses (89 ± 1%, *n* = 101 synapses in 8 experiments, Figures 4A2,B) than blocking P/Q type Ca^2+^ channels (43 ± 3% *n* = 103 synapses in 9 experiments, Figures 4A1,B), as expected from the data in Figure 3. Interestingly, there was a larger range of effects across synapses when we blocked P/Q type channels (Figure 4B, STDEV_P/Qeffect_ = 0.27 compared to STDEV_Neffect_ = 0.14, compare also Figures 5A and 6A).

Having established the average effects of each toxin at individual boutons, we analyzed the data more closely, searching for correlations between the effect of P/Q or N-type blockers and *Pv* or RRP size. As we expected, there was no correlation between the magnitude of inhibition of either toxin and RRP size (Figures 5C1,C2 and 6C1,C2). Similarly, we did not find any correlation between the effect of toxin and *Pv* (Figures 5B1,B2 and 6B1,B2). These results indicate that Ca^2+^ channel subtypes are not differentially distributed on boutons with different synaptic efficacies.

### Malleability of synapses—the cAMP pathway

After studying the variability in *Pv* and RRP size, and its potential relation to Ca^2+^ channel subtypes, we wondered whether the baseline *Pv* or RRP size might influence how synapses would respond to a potentiating stimulus.

One way of altering synaptic strength is to increase intracellular concentrations of cAMP. This activates protein kinase A (PKA) and Epac, the guanine nucleotide exchange factor for the small G protein Rap, leading to increased *Pv* and/or RRP size in many different neuronal preparations (Chavez-Noriega and Stevens, 1994; Trudeau et al., 1996; Chen and Regehr, 1997; Trudeau et al., 1998; Sakaba and Neher, 2001; Kaneko and Takahashi, 2004; Huang and Hsu, 2006; Gekel and Neher, 2008; Yao and Sakaba, 2010). The most detailed and recent of these studies, using the elegant biophysical tools available in the calyx of Held preparation (Yao and Sakaba, 2010), showed that raising cAMP concentration leads to large increases in *Pr* and much smaller increases in RRP size. The larger *Pr* is not due to changes in Ca^2+^ entry but to an increase in vesicle fusogenicity, as assayed with Ca^2+^ uncaging.

To explore this pathway to synaptic potentiation in our system, we applied forskolin (50 μM). We selected forskolin because it activates adenlyl cyclase directly (Insel and Ostrom, 2003), bypassing possible variability in potentiation effects due to variations in cell surface receptors at different synapses. Single AP responses at 2 mM extracellular Ca^2+^ were larger (+125 ± 35%, *n* = 8 experiments, *P* ≤ 0.004, one-sample, one-tailed *t*-test with the null hypothesis μ ≤ 0, *t*-value = 3.58) due to effects on both *Pv* (+85 ± 27%, *P* = 0.008, one-sample, one-tailed *t*-test with the null hypothesis μ ≤ 0, *t*-value = 3.18) and RRP size (+22 ± 6%, *P* = 0.004, one-sample, one-tailed *t*-test with the null hypothesis μ ≤ 0, *t*-value = 3.60).

**Figure 7:**
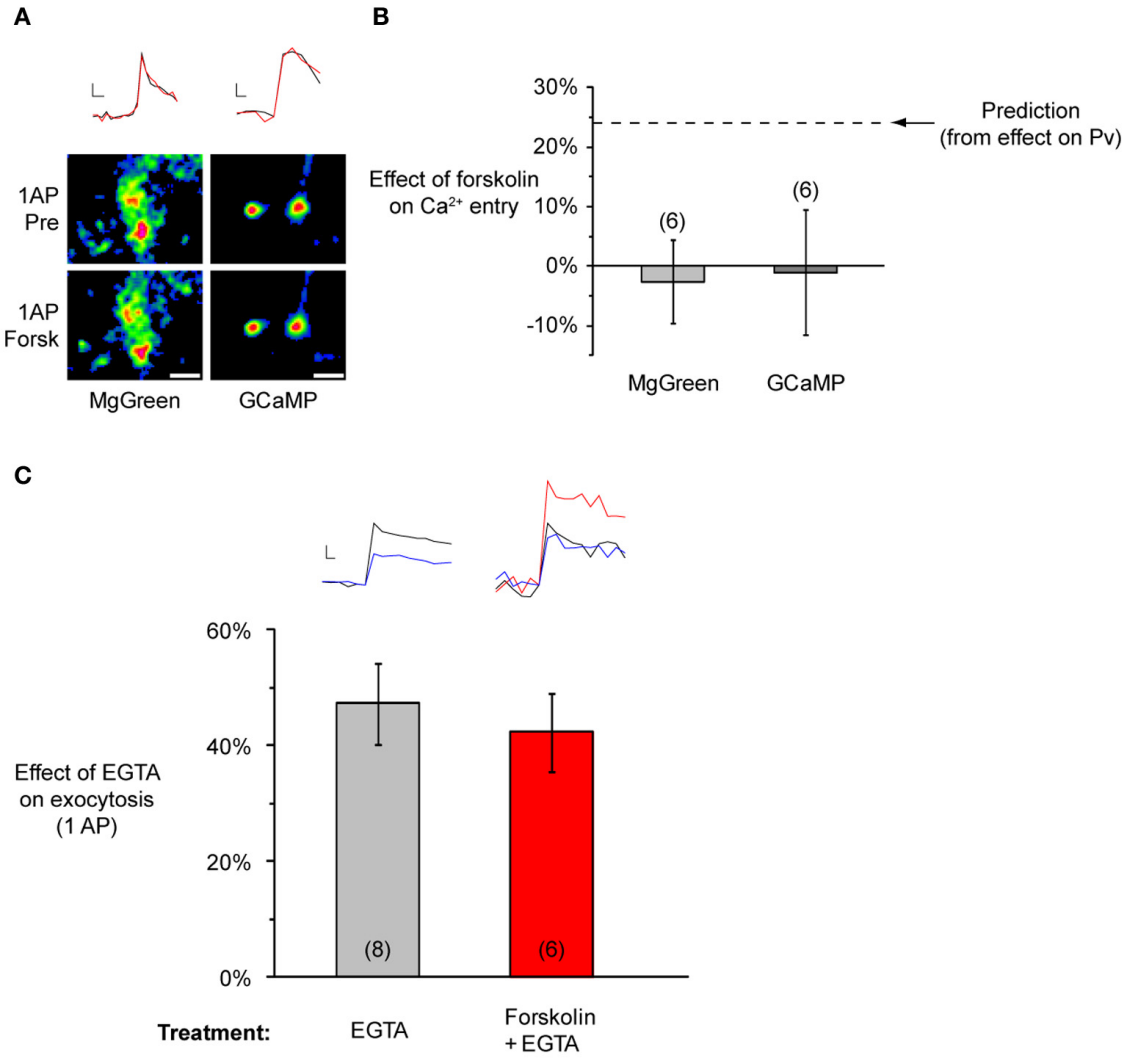
**Forskolin does not affect calcium entry or coupling between Ca^2+^ and primed vesicles. (A)** Representative experiments showing no effect of forskolin on Ca^2+^ entry. *Top:* Sample traces from a MgGreen (left) or GCaMP3 (right) experiment before (black) and after forskolin (red). Scale bar MgGreen: 20% of baseline peak, 25 ms. Scale bar GCaMP3: 20% baseline peak, 50 ms. *Bottom:* Representative responsive regions from each experiment. Each set of images corresponds to a subset of responsive regions included in the analysis. Images are averages of single AP difference images (*n* = 10 trials for MgGreen, *n* = 30 trials for GCaMP3), smoothed for presentation purposes only. Scale bar = 2 μm. **(B)** No effect on single AP Ca^2+^ entry, measured with MgGreen or GCAMP3 with 2 mM extracellular Ca^2+^. The dashed line indicates the predicted effect if forskolin's action on *Pv* were entirely through an increase in Ca^2+^ entry. **(C)** Forskolin does not change the effect of EGTA on single AP exocytosis (EGTA-AM applied for 90 s at 100 μM and washed for 10 min). *Inset at top left:* Sample traces from a single experiment before (black) and after a pulse of EGTA-AM (blue). *Inset at top right:* Sample traces from a single experiment in baseline conditions (black), after forskolin (red), and after adding pulse of EGTA-AM to the forskolin solution (blue). Scale bar MgGreen: 20% of pre forskolin peak, 10 ms.

To clarify the basis of the changes in *Pv*, we studied the effects of forskolin on Ca^2+^ entry in response to a single AP, measured in two ways. First, we used the calcium sensitive dye MgGreen, in its AM-ester form. Second, we used the genetically encoded calcium indicator GCaMP3 (with a correction applied for its non-linear response to Ca^2+^, see Materials and Methods). These methods have complimentary advantages and disadvantages. MgGreen allows high temporal resolution, but it is impossible to completely rule out contributions from postsynaptic compartments [though most of the signal is sensitive to a P/Q and N-type Ca^2+^ channel blocker, see Ariel and Ryan (2010)]. Conversely, using GCaMP3 in sparsely transfected cultures, it is easy to restrict measurements to presynaptic compartments. However, this indicator is comparatively slow, and measurements may confound effects on Ca^2+^ entry and clearance. Using the above measurements of forskolin's effect on *Pv*, and the non-linear relationship between *Pv* and Ca^2+^ entry [Figure 2C in Ariel and Ryan (2010)], we estimated that if the entire effect of forskolin on *Pv* were due to an increase in Ca^2+^ entry, there would be a 24% rise in the latter. However, measurements with MgGreen (−3 ± 7%, *n* = 6, *P* = 0.73 in one-sample, one-tailed *t*-test with the null hypothesis μ = 0, *t-value* = −0.37) and GCaMP3 (−1 ± 11%, *n* = 6, *P* = 0.92 in one-sample, one-tailed *t*-test with the null hypothesis μ = 0, *t-value* = −0.10) showed no such increase (Figures 7A,B). This suggests that forskolin does not raise *Pv* through an increase in Ca^2+^ entry.

Given that forskolin-induced *Pv* increases were not due to larger Ca^2+^ entry, two main possibilities remained to be tested. First, primed vesicles might be more tightly coupled to Ca^2+^ channels. Second, the vesicle fusion machinery might be in a more fusogenic state for any given calcium concentration. The first hypothesis is testable by applying the calcium buffer EGTA (Eggermann et al., 2012). If vesicles move closer to Ca^2+^ channels after forskolin application, then it should be more difficult for EGTA molecules to intercept Ca^2+^ as it diffuses from the channel mouth to calcium sensors on the vesicles. Therefore, we would expect the effect of EGTA on exocytosis to be smaller after applying forskolin. To test this idea, we measured single AP exocytosis before and after applying EGTA-AM (Figure 7C). As expected, this led to a reduction in exocytosis (47 ± 7%, *n* = 8 experiments). However, when we repeated the experiment adding forskolin before the EGTA exposure, the reduction in single AP exocytosis was indistinguishable from the control [42 ± 7%, *n* = 6 experiments, *P* = 0.66 in one-way ANOVA, *F*_(1, 11)_ = 0.21]. This suggests forskolin does not modify the effective distance between vesicles and Ca^2+^ channels. Thus we conclude that forskolin likely acts by making vesicles more fusogenic. A purer test of this would be to artificially control rapid jumps in intracellular calcium using calcium uncaging, something that has only recently become technically feasible in a few preparations (Burgalossi et al., 2010; Trigo et al., 2012). However, our results are completely consistent with those found in the calyx of Held (Yao and Sakaba, 2010), and allow us to speculate that rises in cAMP cause an increase in primed vesicle fusogenicity, without affecting Ca^2+^ entry or vesicle-to-channel coupling.

**Figure 8:**
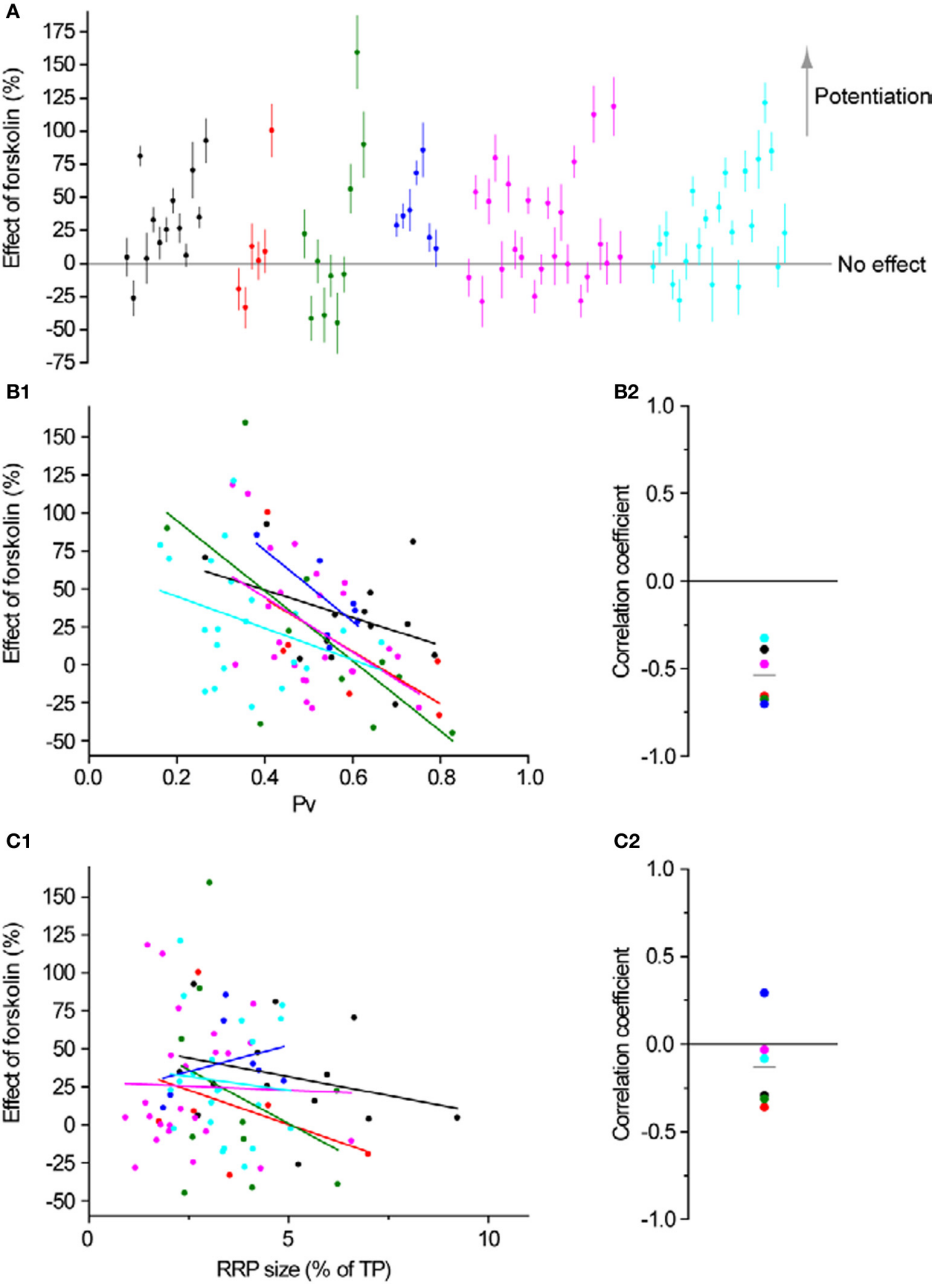
**Synapses are differentially modulated by forskolin according to their baseline *Pv*. (A)** Effect of forskolin on exocytosis in response to a single AP. Different colors represent individual experiments, while each dot shows the effect of the drug on a single synapse with its SE. Experiments are ordered according to their responses to an individual AP from highest (left) to lowest (right). Within an experiment, synapses are also sorted from most (left) to least responsive (right). **(B1,B2)** Effect of forskolin is negatively correlated with *Pv*. **(B1)** Each dot is a synapse and the coloring scheme is the same as in **(A)**. Lines represent best fits for each experiment, colored accordingly. **(B2)** There is a negative correlation between the effect of forskolin and *Pv* (one-sample two-tailed *t*-test against null hypothesis μ = 0 for Pearson correlation coefficients, *P* = 0.0004, *t*-value = −8.17). Each dot represents the value of the correlation coefficient for 1 experiment, with colors consistent with the rest of the figure. The gray line represents the average correlation coefficient across experiments. **(C1,C2)** Effect of forskolin is independent of RRP size. Coloring and symbols are analogous to **(B1)** and **(B2)**. Correlation coefficients of the effect of forskolin with RRP size for each experiment were not significantly different from 0 (one-sample two-tailed *t*-test against null hypothesis μ = 0, *P* = 0.25, *t*-value = −1.31).

All of the aforementioned measurements with forskolin were performed on groups of boutons using 2 mM extracellular Ca^2+^. However, our main goal in using forskolin was to test whether its potentiating effects would be different in synapses with initially different exocytosis properties. To that end, in a subset of the experiments in Figure 2 (6 experiments from 3 independent culture sets), we measured RRP size and *Pv* at single synapses, subsequently applied forskolin and estimated its effect on single AP responses. As expected from a simple model of exocytosis (see Discussion) the effect of forskolin at 4 mM extracellular Ca^2+^ (27 ± 5%, *n* = 81 boutons) was smaller than at 2 mM (+125 ± 35%, *n* = 8 experiments, see above). Interestingly, the effect of forskolin was very variable across synapses (Figure 8A) but showed a clear association with *Pv*. We consistently observed a negative correlation between the effect of forskolin and initial *Pv* (Figures 8B1,B2). Conversely, there was no correlation between the effect of forskolin and RRP size (Figures 8C1,C2). Therefore, synapses with lower basal *Pv* showed larger responses to the forskolin treatment.

**Figure 9:**
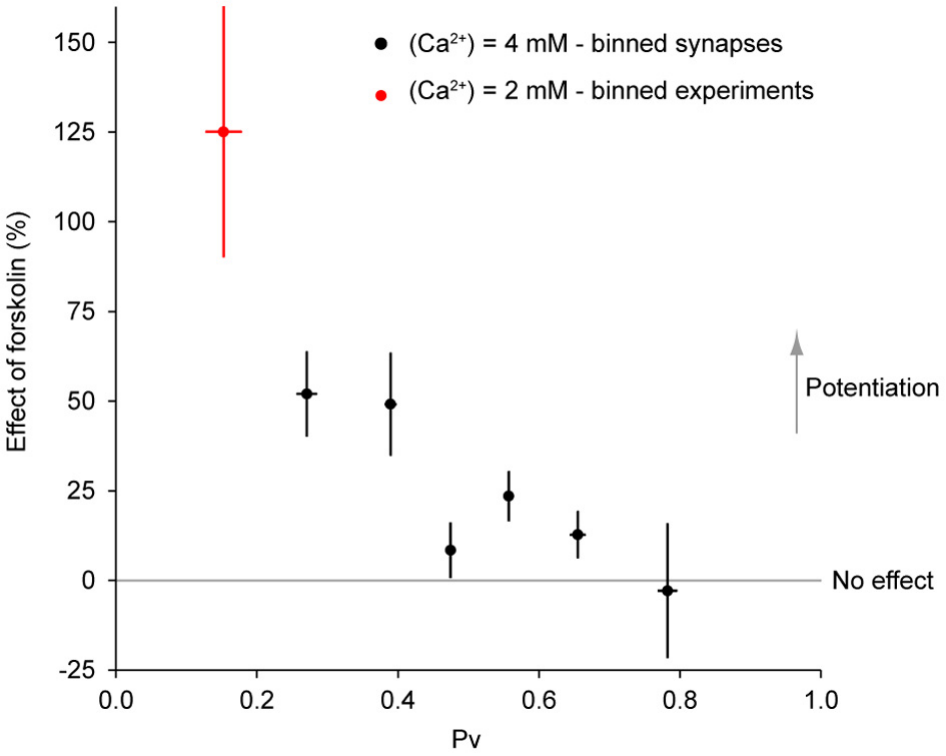
**The effect of forskolin on exocytosis in response to a single action potential is negatively correlated with *Pv*.** Combined data with 4 mM extracellular Ca^2+^ (Figure 8B1, binned across synapses) and 2 mM extracellular Ca^2+^ (see text, binned across experiments) shows an inverse relationship between initial *Pv* and the effect of forskolin (ρ = −0.88, *P* = 0.009).

To explore this relationship further, we binned the data in Figure 8B1 according to initial *Pv*. When the data are thus binned the inverse relationship between the effect of forskolin and initial *Pv* becomes even clearer (Figure 9). Furthermore, by incorporating the data obtained with 2 mM extracellular Ca^2+^, it becomes evident that this general trend also applies to cases where initial *Pv* is smaller because extracellular Ca^2+^ is lower. In the discussion, we present a simple model that can explain these observations.

## Discussion

We present here methods that allow precise estimates of *Pv* and RRP size at the level of individual presynaptic terminals. We find there is great variability between synapses in their baseline *Pv* and RRP size, even when comparing boutons on the same axonal branch. We find that the exact repertoire of Ca^2+^ channel subtypes varies substantially between synapses, but is not predictive of synaptic efficacy. Finally, the potential for modulation by increasing cAMP levels is highly variable between individual presynaptic terminals, but overall follows a simple rule: synapses with lower initial *Pv* can be potentiated more.

### Measurements at individual synapses

Previous reports have shown a large amount of variability in release properties between individual synapses of the same axon. Our study adds to a few others that have begun to systematically estimate the variability in *Pv* across synapses (Granseth and Lagnado, 2008; Ermolyuk et al., 2012). Our work illustrates a problem that is sometimes unrecognized when measuring properties of single AP potential exocytosis in individual small nerve terminals: statistical fluctuations inherent in the process being studied can render estimates of release parameters very imprecise, particularly when *Pr, Pv*, and/or *n* (the number of vesicles in the RRP) are low. This issue is particularly noteworthy because it cannot be sidestepped by improving the quality of the measurement system; rather, it is a consequence of the binomial nature of synaptic vesicle exocytosis. Equation 2 makes explicit how this will affect measurements of *Pv*. As mentioned above the more typically measured parameter is *Pr*, that is, the probability that a synapse will not fail in response to an AP. Using the same logic as for Equation 2, the CV in a measurement of *Pr* based on an experiment with *k* trials will be:

(3) $$\text{CV}=\sqrt{\frac{\left(1-Pr\right)}{k\times Pr}}$$

Thus, assuming *Pr* = 0.3 (a typical value reported with 2 mM extracellular Ca^2+^), to perform experiments where this parameter can be measured with an expected CV of less than 15% will require at least *k* = 88 runs. In addition, these runs need to be temporally spaced such that they are not influenced by short-term plasticity effects. These caveats are worth keeping in mind when designing and interpreting experiments where *Pr* and/or *Pv* are measured at individual synapses, particularly if conditions are such that these parameters are expected to be low. Given that physiological conditions typically involve higher temperatures and lower extracellular Ca^2+^ concentrations than those widely used for experiments in culture (Pyott and Rosenmund, 2002), this will likely lower *Pr* and further exacerbate this measurement challenge. Here, we purposefully shifted our synapses to a higher *Pv* regime and optimized our imaging conditions to ensure cell viability over the course of long experiments. Hopefully, further optimization will allow even more prolonged measurement windows under more physiological conditions.

### Possible determinants of variation in *Pv* and RRP size

The large amount of variability in both *Pv* and RRP size between synapses made by the same axonal branch begs the question of what factors are responsible for these differences. One major determinant is Ca^2+^ entry, which can vary systematically across synapses in a way that accounts for variability in *Pv* and *Pr* (Rozov et al., 2001; Koester and Johnston, 2005; Brenowitz and Regehr, 2007; Ermolyuk et al., 2012; Holderith et al., 2012; Sheng et al., 2012). Recent studies show that a critical control variable regulating Ca^2+^ entry is the number of Ca^2+^ channels at the presynaptic membrane (Holderith et al., 2012; Sheng et al., 2012). However, little is known about how the number of Ca^2+^ channels is set at the level of individual synapses. More generally, proteins that affect not only the number of Ca^2+^ channels, but their unitary currents, or their distribution in the active zone will be promising candidates for study. For example, the recent discovery that alpha2delta strongly affects Ca^2+^ trafficking and function makes it a promising candidate to explain differences between single synapses (Hoppa et al., 2012). Another molecule that has garnered recent attention is Rab3 interacting molecule (RIM), a protein enriched at active zones (Mittelstaedt et al., 2010) which controls *Pv* and RRP size through specific domains that interact directly with munc13 and Ca^2+^ channels, affecting priming, vesicle-to-channel coupling, and fusogenicity (Deng et al., 2011; Han et al., 2011; Kaeser et al., 2011). Additionally, RIM's relatively short half-life (around an hour, Yao et al., 2007) suggests it might act as a dynamic, rate-limiting control variable that sets synaptic strength. Finally, in a recent study, the levels of RIM at individual synapses were correlated with the total uptake of an antibody against a synaptic vesicle protein during stimulation (Lazarevic et al., 2011). While this is a comparatively crude measure of synaptic output, the correlation is nevertheless suggestive. Of course, there are many other molecules that might modulate *Pv* or RRP size and explain their variability between individual synapses. The methods we have developed here should aid in the search for those factors.

### Ca^2+^ channel subtypes

The synapses in our study were more sensitive to block of N than P/Q-type Ca^2+^ channels. The relationship between the suppression of Ca^2+^ influx and the suppression of exocytosis by blockade of each channel was predicted well by the curve relating calcium influx and exocytosis in our system, determined previously (Ariel and Ryan, 2010). This correspondence indicates that both types of channels have similar coupling to release sites (Figure 3). The lack of difference in coupling between Ca^2+^ channel subtypes agrees with previous reports in hippocampal neurons (Wu and Saggau, 1995; Reid et al., 1998). This leaves differing numbers of channels or currents per channel as possible explanations for the larger effect of N-type block. Unitary Ca^2+^ channel properties were recently measured in the calyx of Held synapse and did not differ significantly between N and P/Q subtypes (Sheng et al., 2012). If this holds in our experimental system it would suggest that there are typically more N than P/Q-type Ca^2+^ channels in hippocampal synapses in culture.

An intriguing observation was the lower variance across synapses in the effect of an N-type inhibitor on single AP exocytosis, compared to a P/Q-type inhibitor (Figure 4). Initially, we speculated that some of this might be due to a ceiling effect, given that reductions in single AP responses by blocking N-type channels are close to the 100% bound. If this were the case, we would expect experiments with large average effects of either toxin (i.e., closer to the 100% bound) to have less variability among synapses in the effects of the toxin. However, the standard deviation of the effect of the toxin was not correlated with the average toxin effect for experiments in the presence of N-type (ρ = 0.32, *P* = 0.44, *n* = 8 experiments) or P/Q-type blockers (ρ = 0.35, *P* = 0.35, *n* = 9 experiments). This suggests that a ceiling effect cannot account for the larger variability between synapses in the effect of a P/Q-type Ca^2+^ channel blocker, compared to an N-type blocker.

An alternative explanation for the larger variability among synapses in the effect of a P/Q-type blocker, compared to an N-type blocker, can be found by analyzing the curve that relates exocytosis to Ca^2+^ entry (Figure 3). Since the average effect of P/Q-type blockers is smaller, synapses move down this curve to a region that is steeper than when they are blocked with an N-type blocker. Thus, comparable variance in block of Ca^2+^ entry will translate to larger variance in exocytosis for P/Q vs. N-type Ca^2+^ channel block. Since we did not measure Ca^2+^ entry directly at the level of single synapses we do not know whether this explanation fully accounts for the difference in variance between the effects of P/Q and N-type Ca^2+^ channel blockers, or whether other factors might play a role.

Our results rule out the hypothesis that different Ca^2+^ channel subtypes are associated with synapses of different efficacies. There was no clear relation between a synapse's initial *Pv* and the type of Ca^2+^ channels present. Thus, the relative mix of N and P/Q-type Ca^2+^ channels present at a presynaptic terminal does not affect baseline synaptic strength (in agreement with a previous study in younger, autaptic cultures, Reid et al., 1997). Rather, differences in how these channel subtypes can be modulated (Kisilevsky and Zamponi, 2008) suggest that Ca^2+^ channel repertoire will be critically important to how individual synapses' efficacy can be modified, and to their short-term plasticity properties.

### Potentiation by forskolin at individual synapses

**Figure 10:**
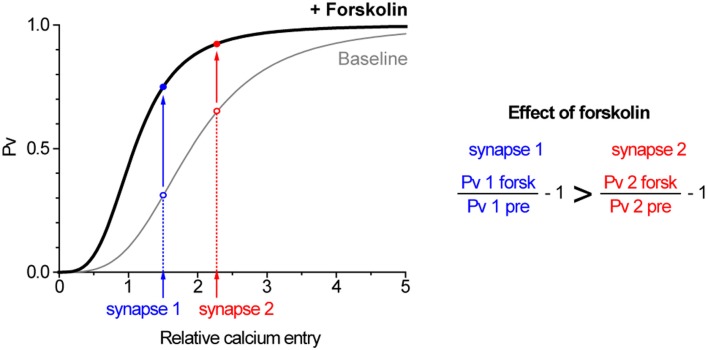
**A simple model can explain how forskolin has a greater effect on synapses with lower *Pv*.** The graph illustrates two synapses with differing Ca^2+^ entry in response to single AP (synapse 2 > synapse 1) in which primed vesicles have the same fusogenicity (gray curve). When forskolin is applied, the fusogenicity of primed vesicles increases equally in both synapses (black curve, shifted left) such that they increase their *Pv* (solid arrows). However, synapse 1, with an initially lower *Pv*, exhibits a larger increase. The baseline curve is the measured Hill relationship between Ca^2+^ and exocytosis (Ariel and Ryan, 2010), whereas the effect of forskolin is modeled as a leftward shift due to a 50% reduction in the Km of that relationship (see Discussion).

An intriguing observation from our study was the negative correlation at the level of individual synapses between the effect of forskolin on single AP responses and *Pv*. To investigate this correlation in more detail we used a few simple models. A first step to study the effect of an activator on boutons with varying initial *Pv* is a model explaining why those synapses are different in the first place. There are two general explanations for *Pv* variation across boutons. The first option is that primed vesicles have different fusogenicities across different synapses. Alternatively, boutons might vary in the relative entry of Ca^2+^ ions in response to 1 AP. As regards forskolin, its effect on *Pv* seems likely to be due to an increase in the fusogenicity of primed vesicles. In Figure 10, we illustrate a hypothetical scenario with two synapses in which initial *Pv* varies due to differential Ca^2+^ entry and forskolin causes a uniform increase in fusogenicity. We base this model on the previously established relationship between Ca^2+^ entry and exocytosis in our system (Ariel and Ryan, 2010) and account for an increased fusogenicity in forskolin as a left-shift in that curve. Under these conditions, forskolin will cause a larger increase in exocytosis in response to 1 AP in the synapse with lower initial *Pv*. We emphasize that in this scenario forskolin causes the same increase in fusogenicity in both boutons. The differential effect on single AP exocytosis arises as a result of the shape of the *Pv* vs. Ca^2+^ entry curve and the initial position on that curve of a single AP at 4 mM in both synapses. An alternative scenario, where initial variation in *Pv* is due to differences in fusogenicity gives similar results, and the addition of forskolin's effects on RRP size do not modify the basic conclusion (not shown). Finally, if we imagine that the two synapses illustrated in Figure 10 are actually two external calcium conditions to which we expose a single synapse, this explains the larger effect of forskolin under conditions of smaller Ca^2+^ entry (Figure 9). Thus, a very simple model accounts well for the general trends seen in our data. While we cannot exclude that the effects of forskolin are not homogenous across boutons, we emphasize that in principle, this is not necessary to obtain non-homogenous effects on *Pv*. In fact, the model suggests that any intervention that uniformly increases the fusogenicity of primed vesicles across synapses will lead to bigger effects at boutons with lower initial *Pv*.

### Potential for future experiments

The methods developed herein can be used to explore many potential determinants of intersynapse variability. Does the concentration of alpha2delta or RIM at a single synapse set *Pv* at that bouton? Do target-specific effects on presynaptic boutons act by modifying *Pv*, RRP size, or both? Are synapses formed by inhibitory and excitatory axons equally variable? Does the proximity of synapses to active astrocytes explain why some synapses are stronger than others (Perea and Araque, 2007)? These are a small sample of the many kinds of questions that can be addressed using our methods.

## Conclusions

Overall, our study of individual synapse properties highlights the large amount of variability present in both basal exocytosis parameters and the degree to which those parameters can be modulated, even for synapses that belong to the same axon. While we cannot rule out that this variability is an artifact of our culture conditions, several examples in more intact preparations suggest it is representative of what actually occurs *in vivo* (Markram et al., 1998; Reyes et al., 1998). If this variability is indeed a defining feature of hippocampal neurons—or neurons in general—it is interesting to speculate whether it is simply a byproduct of cellular processes that use relatively few molecules (Ribrault et al., 2011) or whether, in addition, it has some adaptive value for neural circuit function (Branco and Staras, 2009).

## Materials and methods

### Cell culture and optical setup

We dissected hippocampal CA3-CA1 regions from 1- to 3-d-old Sprague Dawley rats (of either sex), dissociated the cells, and plated them onto poly-ornithine-coated glass as described previously (Ryan, 1999). We transfected cells with a chimera of the pH-sensitive GFP pHluorin and the vesicular glutamate transporter (vG-pH, Voglmaier et al., 2006), or the genetically encoded calcium indicator GCaMP3 (Tian et al., 2009), using calcium phosphate precipitation 6–9 days after plating. Imaging was performed after 13–25 days *in vitro*. Coverslips were mounted in a rapid-switching, laminar-flow perfusion, and stimulation chamber (volume ~75 μl) on the stage of a custom-built laser-illuminated epifluorescence microscope. Live-cell images were acquired with an Andor iXon+ (model #DU-897E-BV) back-illuminated electron-multiplying charge-coupled device camera. An Ar^+^ ion or solid-state diode pumped 488 nm laser was shuttered using acousto-optic modulation. Fluorescence excitation and collection was through a 40 × 1.3 NA Fluar Zeiss objective using 515–560 nm emission and 510 nm dichroic filters (Chroma), and a 1.6× Optivar. Laser power at the back aperture was ~4 mW for imaging of vG-pH and ~1 mW for imaging of GCaMP3 or Magnesium Green (MgGreen). APs were evoked by passing 1 ms current pulses, yielding fields of ~10 V/cm via platinum-iridium electrodes. Experiments analyzing the effects of forskolin with 2 mM external Ca^2+^ were performed at room temperature (~28–32°C in stimulation chamber), for all others the temperature was clamped at 30.0 ± 0.1°C. Cells were continuously perfused at 0.2–1.0 ml/min. in a Tyrode's saline solution containing (in mM) 119 NaCl, 2.5 KCl, 25 HEPES, buffered to pH 7.4, 30 glucose, 10 μM 6-cyano-7-nitroquinoxaline-2,3-dione (CNQX), and 50 μM D,L-2-amino-5-phosphonovaleric acid (AP5). For experiments with 2 mM external Ca^2+^ concentration, the perfusion solution contained (in mM) 2 CaCl_2_, 2 MgCl_2_, whereas for experiments with 4 mM external Ca^2+^ concentration, the solution contained 4 mM CaCl_2_ and no MgCl_2_. All chemicals were obtained from Sigma except for Ca^2+^ channel toxins (Alomone Labs) and MgGreen (Invitrogen). Due to the low baseline fluorescence of neurons that express vG-pH (Balaji and Ryan, 2007), we sometimes gave brief bursts with 6 APs at 33 Hz every 4 s to find transfected cells in a dish. Cells were allowed to rest 10 min after identification with 33 Hz stimuli, at least 45 s between 1 AP trials and at least 5 min between 100 Hz AP bursts. In cases where we equate single experiments with individual neurons, we confirmed this was the case by *post-hoc* immunostaining for vG-pH using an antibody against GFP (Invitrogen) and unambiguously tracing the imaged axons to a single transfected soma.

### Imaging conditions

We optimized our data acquisition integration time and the frequency of exposures according to the parameter we wished to measure. To estimate RRP size, we acquired data at 100 Hz by integrating for 9.74 ms in frame transfer mode and restricting imaging to a subarea of the CCD chip. The maximum width of the imaged field under those conditions was 167 pixels (41.75 μm). For most single AP trials using vG-pH, we used 2 Hz imaging, integrating for 25 ms. This gave us more precision, at the expense of slower imaging rates in those trials. This slower time resolution is still much faster than the fluorescence decay after stimulation due to endocytosis and reacidification, which takes at least several seconds (Armbruster and Ryan, 2011). For experiments evaluating the effects of forskolin with 2 mM extracellular Ca^2+^ we used 100 Hz imaging for single AP runs. Images were analyzed in ImageJ (http://rsb.info.nih.gov/ij/) using a custom-written plugin (http://rsb.info.nih.gov/ij/plugins/time-series.html). 2 μm diameter circular ROIs were placed on all varicosities that did not split or merge, and were stably in focus throughout all trials.

### RRP size estimation

To determine the size of the RRP, we used a slightly modified version of a previously developed protocol (Ariel and Ryan, 2010). Briefly, we stimulated at 100 Hz in 2 mM (experiments with forskolin in the presence of 2 mM Ca^2+^) or 4 mM (all other cases) external Ca^2+^ and then used a semi-automatic method to search for plateaus in the Δ*F* response during stimulation (for example, see Figure 1B1). Sliding data windows of 5 or more APs were used to fit a linear model to the cumulative Δ*F* vs. AP number data. A plateau representing the RRP size was identified as the window where the absolute slope of Δ*F* vs. AP number was the smallest (i.e., the flattest region). To calculate the RRP size, we averaged the Δ*F* values within the identified window. Single AP Δ*F*s were estimated as the point to point difference before and after the stimulus except when the data was acquired at 100 Hz. In that case (experiments with forskolin in the presence of 2 mM Ca^2+^) we took the difference between the average 10 frames before the stimulus and 10 frames after the stimulus to estimate Δ*F*s.

### Single synapse experiments

To measure *Pv* and RRP size at individual boutons (experiments in Figures 1, 2, 5, 6, and 8), we used an optimized protocol that minimized experimental noise and statistical fluctuations yet consistently ensured cell viability. Once a field with transfected synapses was identified, we briefly (<1 min) exposed the preparation to a modified Tyrode's solution buffered at pH = 7.40 with NH_4_Cl instead of HEPES. This solution alkalinizes the interior of all synaptic vesicles and the resulting Δ*F* is proportional to the total number of vG-pH molecules in the terminal. Given that there is typically one vG-pH molecule per synaptic vesicle, and that all vesicles are labeled (Balaji and Ryan, 2007), NH_4_Cl Δ*F* was considered proportional to the total number of vesicles in the synapse (or total pool, TP). The amount of exocytosis (Figures 1B1,B2,C) and RRP size (Figures 1D, 2B1,B2, 5C1, 6C1, 8C1) were therefore expressed as a fraction (or %) of the TP. Following the NH_4_Cl stimulus, we interleaved 30 single AP runs (spaced at least 45 s apart) and 12 trials designed to measure RRP size (where the stimulus was a 20 AP burst at 100 Hz, spaced at least 5 min apart). These runs served as a baseline to estimate *Pv* and RRP size. In a subset of experiments, we added forskolin or specific Ca^2+^ channel blockers to the media and measured the resulting effect on single AP responses in 30 independent trials. We ended each experiment with a final NH_4_Cl application to ensure boutons had not moved, split, merged, or fallen out of focus over the course of the experiment. In total, we initially measured *Pv* and RRP size at 624 individual synapses in 33 experiments from 10 independent culture sets.

We used this data set as a starting point and applied several quality control procedures to ensure synapses had appropriately high signal-to-noise, were stable throughout the experiment and provided a reliable estimate of RRP size. We designed criteria that eliminated synapses with obvious problems (such as instability or poor signal-to-noise) and left us with a high quality data set. In addition, we discarded experiments with less than 5 synapses that fulfilled these criteria.

#### Quality of signal

- Signal-to-noise of 1 AP average response >4
- Standard error (SE) of forskolin or Ca^2+^ channel blocker treatment effect <0.3 (where applicable).

#### Stability of signal

- Linear fit of 1 AP responses as a function of time does not show significant slope (at significance level α = 0.01)
- Linear fit of synchronous response to 20 APs (delivered in 100 Hz bursts) as a function of time does not show significant slope (at significance level α = 0.01).

#### RRP size determination

- In a few cases, there was not a strong reduction in exocytosis rates during the 100 Hz bursts designed to estimate RRP size. We wished to exclude these synapses where the RRP estimate would likely require a substantial correction for ongoing priming. To that end, we defined a variable that estimated the residual RRP recovery (repriming and exocytosis) during the detected plateau, and filtered our synapses according to that variable. As a measure of residual RRP recovery we took the product of the width of the plateau and the slope of a line fit in that region. We only accepted cases where there was, at most, a 15% recovery of the RRP during the plateau detected by our algorithm. In our final data set, the average recovery was 3.2% of the RRP size, indicating there is little contamination of our RRP estimates by priming. Note that this filter criterion does not apply to repriming and exocytosis *after* the detected plateau (for example, see Figure 1B1, exocytosis for APs 10 and higher). As mentioned in our previous manuscript (Ariel and Ryan, 2010), we interpret this as the RRP refilling process “catching up” due to the elevated Ca^2+^ inside the terminal, and generating newly primed vesicles that rapidly fuse with the membrane.

A total of 410 synapses in 32 experiments (66%) passed all criteria and formed the basis for further analysis. We examined whether the quality control filtering might bias our data in any way. Even before the filtering criteria were applied, we noted a skew toward more responsive synapses than expected, evidenced by a higher *Pv* (0.405 ± 0.008) compared to our previous average at 4 mM extracellular Ca^2+^ (0.35, Ariel and Ryan, 2010). We speculate this was due to an implicit bias toward selecting more responsive boutons for single synapse experiments. In addition, after applying our filtering criteria we were left with an even more responsive subset of synapses (*Pv* =0.440 ± 0.008). This is unsurprising given that the filtering criteria include a signal-to-noise cut-off, which will be correlated with *Pv*. Thus, our experiments to study single synapse properties came from a population of boutons with higher *Pv* than those in other studies of exocytosis using vG-pH in our lab (Ariel and Ryan, 2010; Hoppa et al., 2012).

Forskolin was applied continuously at 50 μM. All measurements began at least 5 min after the start of incubation. Its effects on single AP exocytosis (Figures 8A,B1, and C1), *Pv* and RRP size (see text), or Ca^2+^ entry (Figures 7A,B) were calculated as Forsk/Pre-1. EGTA-AM was applied for 90 s at 100 μM, and allowed to wash-off for 10 min before measuring its effect on exocytosis. Its effect on single AP exocytosis (Figure 7C) was calculated as 1-EGTA/Pre.

To block P/Q-type Ca^2+^ channels we applied ω-agatoxin IVA (400 nM) for 2 min. To block N-type Ca^2+^ channels we applied ω-conotoxin GVIA (1 μM) for 2 min. Neither toxin showed any sign of wash-off during prolonged experiments. Toxin effects on single AP exocytosis (Figures 5A,5B1,5C1, 6A,B1, and C1) were calculated as 1-Toxin/Pre.

### Imaging conditions and data analysis of Ca^2+^ indicator experiments

To measure Ca^2+^ influx during single APs using MgGreen (Figures 7A,B, left), the indicator was loaded at 20 μM in its acetoxymethyl ester (AM) form for 10 min and washed-off for at least 10 min before imaging started. Single AP stimuli led to robust, focal responses distributed over neuritic fields. We analyzed Δ*F*/*F*_0_ of manually drawn ROIs placed on these punctate responsive regions, taking care to avoid somas. *F*_0_ was corrected point to point by subtracting local background from manually drawn ROIs on adjacent non-responsive regions. Imaging was performed at 100 Hz and the peak Δ*F*/*F*_0_ in response to a single AP was taken as the difference between the peak and the last 10 frames before the stimulus. To examine the effect of forskolin, responses were averaged across 10 trials before and after drug application. To examine the effect of Ca^2+^ channel blockers, responses were averaged across 6 trials before and 12 trials after drug application. In a few MgGreen experiments, there was a slight rundown in responses (~1% per minute). To correct for this effect, we fit a straight line to the responses in the absence of the drug as a function of time. We subsequently estimated the decrease expected from this rundown in drug trials and corrected the measurement of 1 AP Δ*F*/*F*_0_ in forskolin accordingly. When necessary, these corrected values were used to estimate the effect of the drug on single AP Ca^2+^ responses.

To measure Ca^2+^ increases after stimulation using GCaMP3, we imaged at 20 Hz, integrating for 19.70 ms during each frame (Figures 7A,B, right). The response to a single AP was calculated as the point to point difference before and after the stimulus. Responses were averaged across 30 trials before and after forskolin application. To normalize responses, at the end of each GCaMP3 experiment we exposed neurons to 200 μM ionomycin, which allows full Ca^2+^ exchange across the plasma membrane. The resulting maximal signal was used to normalize traces (Hoppa et al., 2012):

$$F_{\text{norm}(\text{t})}=\frac{F_{\left(t\right)}-F_{\left(0\right)}}{F_{\text{max}}-F_{\text{dark}}}$$

where *F*_(0)_ is the last point before stimulation, *F*_max_ is the maximal value reached during application of ionomycin and *F*_dark_ is the baseline fluorescence of our detector in the absence of illumination. This normalization allowed us to compare experiments with varying amounts of the indicator. GCaMP3 responses are non-linear with respect to Ca^2+^ in the range of interest (Tian et al., 2009) and must be linearized after normalization. To that end, a calibration curve was constructed (Hoppa et al., 2012) by measuring responses to 1–20 APs at 100 Hz, in 2 mM extracellular Ca^2+^ using both MgGreen—known to be a linear indicator in this range (Ariel and Ryan, 2010)—and GCaMP3. The resulting GCaMP3 vs. MgGreen data was fit to a generalized Hill equation. Using that equation and the optimized fit parameters, we could convert any normalized GCaMP3 Δ*F* to a linearized MgGreen equivalent, proportional to the actual ΔCa^2+^. These linearized values were then used to estimate the effect of forskolin on single AP Ca^2+^ responses.

### Statistical analysis

All values mentioned in the text are averages ± standard errors of the mean (SE) unless stated otherwise. All error bars in graphs are SEs unless stated otherwise.

Parametric statistics were used whenever possible (Sokal and Rohlf, 1994). To test the effects of forskolin treatment on single AP exocytosis, *Pv*, RRP size, and Ca^2+^ entry (as defined above), we performed one-sample *t*-tests using the null hypothesis μ = 0. Normality was assayed in all cases using the Shapiro-Wilk test, using α = 0.05 as a cut-off. When an ANOVA was used, the assumption of homogeneity of variances was assayed with Levene's test, using α = 0.05 as a cut-off. Both assumptions were met in all cases. *t*-tests were performed in Excel (Microsoft), Shapiro–Wilk, Levene, and ANOVAs were performed in STATISTICA 8 (StatSoft). The effects of forskolin on all parameters were also evaluated using paired *t*-tests comparing each measured parameter before and after forskolin application and gave the same results (not shown).

To test whether there were correlations between the effect of a drug (forskolin, or Ca^2+^ channel blockers) and either *Pv* or RRP size, we used one-sample, two-tailed *t*-tests on the correlation coefficients with the null hypothesis μ = 0.

### Error analysis

To estimate how precisely we could determine *Pv* and RRP size at individual synapses (Figures 1D,E), we extended the same formulas we had previously used to estimate errors in *Pv* and RRP size within cells (Ariel and Ryan, 2010). Our analysis is based on a SE propagation formula (Taylor, 1997):

if q≡q(x,…,z)

then

$$\delta q=\sqrt{{\left(\frac{\partial q}{\partial x}\delta x\right)}^{2}+\cdots +{\left(\frac{\partial q}{\partial z}\delta z\right)}^{2}}$$

Briefly, we relied on 3 traces from each bouton:

*F*_1_: response to 1 AP (average of 30 trials)
*F*_20_: response to 20 APs at 100 Hz (average of 12 trials)
*F*_NH_4_Cl_: response to a pulse of 50 mM NH_4_Cl, which alkalinizes all cellular compartments.
*F*_1_ traces were acquired at 2 Hz and we calculated Δ*F*_1_ as the difference between the last point before firing an AP and the first point after the stimulus. To estimate SE_Δ*F*_1__ we took the SE of the response to 1 AP across 30 trials.

We estimated SE_NH__4_Cl from the SE of the baseline and peak fluorescence during NH_4_Cl application:

$$\begin{array}{l} \;\Delta F_{{\text{NH}}_{4}\text{Cl}}=F_{{\text{NH}}_{4}\text{Clpeak}}-F_{{\text{NH}}_{4}\text{Clpre}},\\ {\text{SE}}_{\Delta F_{{\text{NH}}_{4}\text{Cl}}}=\sqrt{{\text{SE}}_{F_{{\text{NH}}_{4}\text{Clpeak}}}^{2}+{\text{SE}}_{F_{{\text{NH}}_{4}\text{Clpre}}}^{2}} \end{array}$$

where the SE was the standard deviation of the 10 first frames divided by the square root of 10 and of the *i* frames of the peak divided by the square root of *i*.

For the 20 AP traces we proceeded similarly, averaging the last 10 frames before the stimulus and the frames included in the plateau to obtain:

$$\begin{array}{l} F_{\text{20}\;\text{pre}},{\text{ SE}}_{F_{\text{20}\;\text{pre}}}\\ F_{\text{20}\;\text{plateau}},{\text{ SE}}_{F_{20\;\text{plateau}}} \end{array}$$

where the SE was the standard deviation divided by the square root of the number of observations in each case (10 for *F*_20 pre_ and the number of points included in the plateau for *F*_20 plateau_). Thus:

$$\begin{array}{l} \;\Delta F_{\text{20}\;\text{plateau}}=F_{\text{20}\;\text{plateau}}-F_{\text{20}\;\text{pre}},\\ {\text{SE}}_{\Delta F_{\text{plateau},\text{inst}}}=\sqrt{{\text{SE}}_{F_{\text{20}\;\text{plateau}}}^{2}+{\text{SE}}_{F_{\text{20}\;\text{pre}}}^{2}} \end{array}$$

In addition to these instrumental errors, given that we measured the responses to 20 APs at 100 Hz 12 times in each experiment we also obtained a statistical estimate of the error in Δ*F*_20 plateau_:

$${\text{SE}}_{\Delta F_{20\;\text{plateau},\text{stat}}}=\frac{{\text{SD}}_{\Delta F_{20\;\text{plateau},\text{stat}}}}{\sqrt{12}}$$

where SD_Δ*F*__20 plateau,stat_ is the standard deviation of the plateau estimates in different trials. We added the instrumental and statistical contributions to the error in quadrature and combined them to get the total error for Δ*F*_20 plateau_:

$${\text{SE}}_{\Delta F_{\text{20}\;\text{plateau}}}=\sqrt{{\text{SE}}_{\Delta F_{20\;\text{plateau},\text{inst}}}^{2}+{\text{SE}}_{\Delta F_{20\;\text{plateau},\text{stat}}}^{2}}$$

Finally, we calculated RRP size and *Pv* with their associated errors:

$$\begin{array}{l} \text{RRP}=\frac{\Delta F_{\text{20}\;\text{plateau}}}{\Delta F_{{\text{NH}}_{4}\text{Cl}}},\;{\text{SE}}_{\text{RRP}}=\frac{1}{\Delta F_{{\text{NH}}_{\text{4}}\text{Cl}}}\sqrt{{\text{SE}}_{\Delta F_{\text{20}\;\text{plateau}}}^{2}+{\text{RRP}}^{\text{2}}{\text{SE}}_{\Delta F_{{\text{NH}}_{\text{4}}\text{Cl}}}^{\text{2}}}\\ \;Pv=\frac{\Delta F_{1}}{\Delta F_{\text{20}\;\text{plateau}}},\;{\text{SE}}_{Pv}=\frac{1}{\Delta F_{\text{20}\;\text{plateau}}}\sqrt{{\text{SE}}_{\Delta F_{1}}^{2}+Pv^{2}{\text{SE}}_{\Delta F_{\text{20}\;\text{plateau}}}^{2}} \end{array}$$

These calculations provide the error bars for *Pv* and RRP size in individual synapses (Figures 1D,E).

To estimate the errors of the effects on single AP responses of Ca^2+^ channel toxins (Figures 5A and 6A), we first calculated the error in the Δ*F*_1post_ (after each pharmacological treatment) using the same formulas as for Δ*F*_1_ and then used:

$$\text{Effect}=1-\frac{\Delta F_{1\text{post}}}{\Delta F_{1}},{\text{SE}}_{\text{Effect}}=\frac{1}{\Delta F_{1}}\sqrt{{\text{SE}}_{\Delta F_{1\text{post}}}^{2}+{\left(1-\text{Effect}\right)}^{2}{\text{SE}}_{\Delta F_{1}}^{2}}$$

To estimate the errors of the effects on single AP responses of forskolin (Figure 8A), we used:

$$\text{Effect}=\frac{\Delta F_{\text{1post}}}{\Delta F_{1}}-1,\;{\text{SE}}_{\text{Effect}}=\frac{1}{\Delta F_{1}}\sqrt{{\text{SE}}_{\Delta F_{\text{1post}}}^{\text{2}}+{\left(1+\text{Effect}\right)}^{2}{\text{SE}}_{\Delta F_{1}}^{2}}$$

### Conflict of interest statement

The authors declare that the research was conducted in the absence of any commercial or financial relationships that could be construed as a potential conflict of interest.
